# Considering Climatic Factors, Time Lag, and Cumulative Effects of Climate Change and Human Activities on Vegetation NDVI in Yinshanbeilu, China

**DOI:** 10.3390/plants12183312

**Published:** 2023-09-19

**Authors:** Sinan Wang, Xiaomin Liu, Yingjie Wu

**Affiliations:** 1 Yinshanbeilu National Field Research Station of Desert Steppe Eco-Hydrological System, China Institute of Water Resources and Hydropower Research, Beijing 100038, China; 2 Water Conservancy and Civil Engineering College, Inner Mongolia Agricultural University, Hohhot 010018, China

**Keywords:** NDVI, climatic factor, path analysis, human activity, time lag and cumulative effects, remote sensing, dryland

## Abstract

Climate and human activities are the basic driving forces that control and influence the spatial distribution and change of vegetation. Using trend analysis, the Hurst index, correlation analysis, the Moran index, path analysis, residual analysis, and other methods, the effects of human activities and climate factors on vegetation change were analyzed. The results show that: (1) The research area’s normalized difference vegetation index (NDVI) exhibited a substantial upward trend from 2001 to 2020, increasing at a rate of 0.003/a, and the vegetation cover was generally healthy. The generally constant NDVI region made up 78.45% of the entire area, and the grassland, cultivated land, and forest land showed the most visible NDVI aggregation features. (2) The Vegetation is mainly promoted by water and heat, particularly precipitation, have a major impact on plants, with the direct influence of precipitation on vegetation growth being much greater than the indirect effect through the temperature. (3) The trend of NDVI residuals showed obvious spatial variability, presenting a distribution characteristic of high in the south and low in the north. The results of this study can provide a basis for the scientific layout of ecological protection and restoration projects in the Yinshanbeilu area.

## 1. Introduction

Vegetation is a comprehensive indicator of ecological change, with the role of connecting ecological elements such as the atmosphere, soil, and hydrology, which can provide a strong guarantee for natural ecosystems and human production and life [1,2,3]. Regional vegetation change is a result of climate and human activities, both of which can have positive and negative effects on vegetation change [4,5,6]. Temperature and precipitation are the key factors affecting vegetation growth and development by regulating plant photosynthesis, respiration, and soil organic carbon decomposition through the effective cumulative temperature and available moisture, which in turn affects plant growth and distribution [7,8,9]. In addition, human activities can also inhibit or promote vegetation growth [10,11,12]. Therefore, studying the spatial and temporal variation patterns of vegetation cover and determining its relationship with climate change and human activities play an important role in regulating the ecosystem and improving the ecological environment.

Previous studies based on NDVI data have explored the spatial change characteristics of vegetation [13], drivers of vegetation change [14], vegetation impacts on climate [15], and the relationship between NDVI and vegetation health [16]. Methodologically, the studies were mainly carried out using multiple regression residual analysis [17], trend analysis [18], aridity index calculation [19], geodetection [20], assessing the impact of prioritization [21], and color map sharpening [22]. For example, Guo et al. [23] and Jia et al. [24] utilized NDVI data to explore the inter-annual variability of different vegetation types in relation to precipitation and temperature. Wang et al. [25] used variational mode decomposition (VMD) methods to study Inner Mongolia to explore the cyclic variations of NDVI, temperature, and precipitation, and found that NDVI and precipitation had a good cyclic consistency, and the vegetation NDVI was significantly positively correlated with temperature and precipitation before and after removing the interference of cyclic variables. Xu et al. [26] used correlation analysis to explore the driving mechanism of vegetation cover changes and climate factors on both. Chen et al. [27] utilized geodetection to quantify annual precipitation and soil type as the main drivers of vegetation NDVI, with an explanation rate of more than 22%. The study of Chen et al. [28] pointed out that vegetation change and climate in Alxa League showed a weak positive correlation, and vegetation change was impacted to a greater degree by precipitation than temperature. Previous findings have pointed out that vegetation cover change is the main controlling factor causing climate change in the response feedback process, but the pathways and physical mechanisms of its influence on climate are still not clear enough, in addition to the time lag and cumulative effects being rarely considered, which leads to increased uncertainty in the results describing the influence of climate-influencing elements on the change in vegetation growth. In addition to climatic factors, human activities are also important drivers of changes in vegetation cover [29]. Recent studies have shown that global vegetation changes are mainly caused by human activities [30,31], in which China and India have played an important role in global terrestrial vegetation changes through afforestation and improved agricultural efficiency [32]. Cai et al. [33] found that human activities have both positive and negative effects on vegetation cover, and the project of returning farmland to forests and grasslands has greatly contributed to the degree of vegetation cover in the region, while urban expansion, indiscriminate logging, and overgrazing have had a destructive effect on vegetation. In the study of grassland vegetation change in Inner Mongolia, temperature and precipitation are considered to be the main climatic factors leading to changes in grassland vegetation, while other human activities (such as land-use change and afforestation) caused by grazing and changes in population size are considered to be the main anthropogenic factors leading to changes in grassland vegetation. In addition, over-utilization of grasslands, such as grassland reclamation, overgrazing, and mining, are also disturbing factors in the growth of vegetation in the region, and these anthropogenic disturbing forces exceed the ability of the ecosystems themselves and external governance to repair them, which ultimately leads to grassland ecosystems being more fragile than those in other regions. Shen et al. [34] expressed the view that the expansion of arid zones and the tendency of soil moisture to dry out in deeper soils could accelerate mining-induced groundwater depletion under a warming climate. Such accelerated depletion would further exacerbate groundwater scarcity and pose a clear risk of degradation to dryland vegetation. Hanchen et al. [35] concluded that an appropriate increase in grazing intensity may have stimulated the growth of grassland plants and increased plant biomass, which in turn contributed to the increase in grassland NDVI. Therefore, both human activities and climate change can influence vegetation changes and may lead to significant differences in vegetation cover changes in different regions. In Inner Mongolia, we have achieved fruitful results on the impacts of regional climate change on vegetation change and the response of vegetation change to climate change, etc. Most of the climate factors considered in the existing studies are temperature and precipitation, ignoring the impacts of human activities on vegetation growth, and failing to quantitatively separate the impacts of climate change and human activities on vegetation change; however, there is no relevant study in Yinshanbeilu.

In past studies, the relationship between vegetation and hydrothermal factors is generally expressed by a correlation coefficient or partial correlation coefficient, and the expression of their response relationship is mostly based on the provincial, geomorphological, or climatic regions, but the correlation coefficient only reflects the surface linkage of the variables, and the partial correlation coefficient expresses the net correlation relationship between specific variables, in contrast to trail analysis, which has the advantage of being able to analyze the direct and indirect effects of a single factor on the dependent variable. In addition, as Yinshanbeilu is an important ecological barrier in the northern part of China, and its water resources are scarce and its ecological environment is fragile, elucidating the effects of the changes in the vegetation cover by climatic change is very necessary.

Overall, the objectives of this study were to (1) analyze the spatial and temporal trends of climatic factors (temperature and precipitation) and vegetation greenness (characterized by NDVI); (2) differentiate between direct, indirect, and combined impacts of climatic factors on vegetation NDVI; and (3) quantify the lag effect and cumulative effect of meteorological factors on vegetation NDVI and the contribution of human influence to vegetation NDVI. This will help to better understand the intrinsic mechanism of the impacts of climate change and human activities on vegetation changes and will be of great significance in guiding regional vegetation restoration planning and management measures.

## 2. Materials and Methods

### 2.1. Study Area

**Figure 1:**
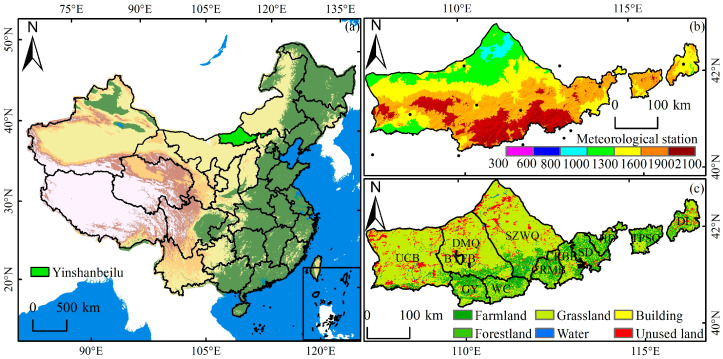
Geographic location of the study area. (**a**) Yinshanbeilu location, (**b**) digital elevation model, and (**c**) land-use type. UCB: Urad central banner, DMQ: Damaoqi, BYEB: Baiyunebo, GY: Guyang, WC: Wuchuan, SZWQ: Siziwangqi, CRMB: Chahar right middle banner, CRBB: Chahar right back banner, SD: Shangdu, HD: Huade, TPSQ: Taipusiqi, DL: Duolun.

Yinshanbeilu in Inner Mongolia is located in the transition zone between the Yinshan Mountain Range and the Mongolian Plateau. The geographical coordinates are 107°17′~116°53′ East longitude and 40°43′~43°23′ North latitude. The administrative scope involves 12 banner counties, with a total area of 97,250.5 km^2^. The terrain of the study area is gradually low from south to north, with an altitude of 941~2295 m. The area is a semi-arid continental monsoon climate in the middle temperate zone, with average annual precipitation of 200~400 mm, average annual temperature of 1.3~3.9 °C, average annual evaporation of 1748~2300 mm, and an average annual frost-free period of 102~121 days. Ecological and environmental problems are becoming increasingly serious (Figure 1).

### 2.2. Data Sources

The vegetation remote sensing data, MODIS NDVI, used in this paper comes from the MOD13A3 product released by NASA, with a time range of 2001–2020 and a spatial resolution of 1 km. In order to eliminate the effects of missing values, water, clouds, heavy aerosols, and cloud shadows on the experimental results, this paper adopts the maximum synthesis method to obtain the annual vegetation NDVI maximum value time series.

Using the meteorological data provided by the National Meteorological Science Data Center of China (http://data.cma.cn/, accessed on 1 June 2023), this paper selects the month-by-month precipitation and month-by-month average temperature data from 12 meteorological stations from 2001 to 2020 and interpolates the precipitation and temperature data into raster data with a resolution of 1 km, based on the inverse distance-weighted averaging method.

### 2.3. Methods

#### 2.3.1. Trend Analysis

Estimating the temporal trend of NDVI using the linear tendency, with the change over time, NDVI often shows the overall upward or downward trend of the series and the change in the spatial distribution pattern [36]. By applying the trend analysis method to analyze the inter-annual trend of NDVI from 2001 to 2020, the formula based on the image element is as follows:

(1) $$\theta_{\mathrm{Slope}}=\frac{n\times \sum_{i=1}^{n}i\times NDVI_{i}-\sum_{i=1}^{n}i\times \sum_{i=1}^{n}NDVI_{i}}{n\sum_{i=1}^{n}i^{2}-{\left(\sum_{i=1}^{n}i\right)}^{2}}$$

where θ*_slope_* is the slope of regression; *n* is the total number of years monitored; and *i* is the time variable.

#### 2.3.2. Hurst Index

The Hurst index can be used to predict the future change trend of long-term time series data relative to the past and is a common method to describe the persistence of the change trend of time series [37]. A Hurst index value, H, between 0 and 1, when 0.5 < H < 1, indicates that the time series future and past show the same trend of change, with continuity. The closer the H value to 1, the stronger the continuity; when H = 0.5, it indicates that the future trend of the time series has nothing to do with the past, an independent random sequence. When 0 < H < 0.5, this indicates that the future and past of the time series show the opposite trend of change, with anti-continuity, and when H is closer to 0, the stronger the anti-continuity. When 0 < H < 0.5, it indicates that the future of the time series and the past show opposite trends, with anti-persistence, and the closer H is to 0, the stronger the anti-persistence.

#### 2.3.3. Coefficient of Variation

The coefficient of variation is used to represent the fluctuation degree of geographical data [38], which can be used to indicate the growth status of grassland, to a certain extent. Its calculation formula is as follows:

(2) $$CV_{NDVI}=\frac{1}{\bar{x}}\sqrt{\frac{1}{\left(n-1\right)}\sum_{i=1}^{n}{\left(x_{i}-\bar{x}\right)}^{2}}$$

where *CV_NDVI_* is the variation coefficient of NDVI; *i* is the time series; *x_i_* is the NDVI of year *i*; and $\bar{x}$ is the mean of multi-year NDVI.

#### 2.3.4. Moran’s I

Moran’s I, as a spatial autocorrelation coefficient, reflects the correlation of the same variable in different spatial locations and is a quantitative indicator of the degree of aggregation of attribute values of spatial units [39], which is calculated as follows:

(3) $$I_{M}=\frac{\sum_{i=1}^{n}\sum_{j=1}^{n}w_{ij}\left(x_{i}-\bar{x}\right)\left(x_{j}-\bar{x}\right)}{S^{2}\sum_{i=1}^{n}\sum_{j=1}^{n}w_{ij}}$$

where *I_M_* is the Moran index; *W_ij_* is the spatial weight between elements *i* and *j*; *x* is the variable; is the mean of the variable; and *S*^2^ is the variance of the variable.

#### 2.3.5. Path Analysis

The correlation between an independent variable and the dependent variable can be divided into two parts, namely, the direct effect of the independent variable on the dependent variable when the effect of other independent variables is not taken into account and the indirect effect of this independent variable on the dependent variable through other independent variables [40]. The pathway analysis method can distinguish between the direct and indirect effects of the independent variable on the dependent variable, which is based on the following principles:

For an interrelated system, if there is a linear relationship between *n* independent variables, *x_i_* (*i* = 1, 2,…, *n*), and a dependent variable, *y*, the regression equation is:

(4) $$y=a_{0}+a_{1}x_{1}+a_{2}x_{2}+\cdot \cdot \cdot +a_{n}x_{n}$$

Based on the simple correlation coefficients, *r_xi xj_*(*i*, *j* ≤ *n*), between the respective variables, and the simple correlation coefficients, *r_xiy_*(*i* ≤ *n*), between the respective variables and the dependent variable, the regular matrix equations can be established by mathematical transformations from Equation (4):

(5) $$\left[\begin{array}{cccc} 1 & r_{x_{1}x_{2}} & \cdot \cdot \cdot & r_{x_{1}x_{n}}\\ r_{x_{2}x_{1}} & 1 & \cdot \cdot \cdot & r_{x_{2}x_{n}}\\ \vdots & \vdots & \ddots & \vdots \\ r_{x_{n}x_{1}} & r_{x_{n}x_{2}} & \cdot \cdot \cdot & 1 \end{array}\right]\left[\begin{array}{c} a_{1}\\ a_{2}\\ \vdots \\ a_{n} \end{array}\right]=\left[\begin{array}{c} r_{x_{1}y}\\ r_{x_{2}y}\\ \vdots \\ r_{x_{n}y} \end{array}\right]$$

In order to facilitate the comparison of indicators with different units and magnitudes, the data were first standardized for NDVI, precipitation, and mean air temperature using the Z-score standardization method:

(6) $$X_{std}=\frac{x_{i}-\bar{x}}{\sigma }$$

where: *X_std_* is the standardized value; $\bar{x}$ is the mean value; and σ is the standard deviation.

Multiple linear regressions were performed on the standardized NDVI (*NDVI_stad_*), mean temperature (*Tmp_stad_*), and precipitation (*Pre_stad_*):

(7) $$NDVI_{stad}=A_{p}\times Pre_{stad}+A_{t}\times Tmp_{stad}+\varepsilon$$

where ε is the residual; and *A_p_* and *A_t_* denote the standardized regression coefficients (direct pathway coefficients) of precipitation and mean temperature on NDVI, respectively, i.e., the direct effect of precipitation and temperature on NDVI when the effects of other variables are not considered.

Due to the interaction between precipitation and mean temperature, the interaction can be expressed as:

(8) $$A_{pt}=r_{pt}\times A_{p}$$

(9) $$A_{tp}=r_{pt}\times A_{t}$$

where *r_pt_* denotes the correlation coefficient between precipitation and mean temperature; and *A_pt_* and *A_tp_* denote the indirect pathway coefficients of precipitation and mean temperature on NDVI, respectively, i.e., the indirect effect of precipitation and temperature on NDVI through another variable.

According to the principle of pass-through analysis, the correlation coefficient is equal to the sum of the direct pass-through coefficient and the indirect pass-through coefficient, that is:

(10) $$r_{p\text{-}ndvi}=A_{p}+A_{pt}$$

(11) $$r_{t\text{-}ndvi}=A_{t}+A_{tp}$$

where *r_p-ndvi_* and *r_t-ndvi_* denote the correlation coefficients of precipitation, mean temperature, and NDVI, respectively, i.e., the combined effect of precipitation and mean temperature on NDVI.

#### 2.3.6. Time Lag and Accumulation Effects of Climatic Variables on Vegetation Change

In order to investigate the time lag effect of NDVI on the response of each climate factor and the cumulative lag effect of NDVI on precipitation [41], the following equations were used, respectively.

(12) $$Pre_{t\left(m,n\right)}=\frac{1}{n}\sum_{i=0}^{n}Pre_{t\text{-}m\text{-}i}$$

(13) $$Tmp_{t\left(m,n\right)}=\frac{1}{n}\sum_{i=0}^{n}Tmp_{t\text{-}m\text{-}i}$$

where *Pre_t(m,n)_* and *Tmp_t(m,n)_* are the *Pre* and *Tmp* of the t month with m months lag and n months accumulation, calculated as the average of the *Pre* and *Tmp* of the lag month and the nth month before the lag month; m is the number of months lag; n is the number of months accumulation; and *Pre_t-m-i_* and *Tmp_t-m-i_* denote the *Pre_(m+i)_* and *Tmp* of the *t* month prior to the *t* month (*m* + *i*) months before month t.

#### 2.3.7. Residual Analysis

Multiple linear regression residual analysis was used to distinguish the effects of climate change and non-climatic factors such as human activities on the changes in NDVI. First, a linear regression model was constructed with temperature and precipitation as independent variables and NDVI as the dependent variable, and the model parameters were calculated Then, based on the temperature and precipitation data as well as the constructed regression model, the predicted value of NDVI (*NDVI_pre_*) was calculated, and the trend rate (*Slope_pre_*) was used to express the effect of climate change on the trend of NDVI; finally, the difference between the observed value of NDVI (*NDVI_i_*) and $NDVI_{i}^{\prime }$ was calculated, and the residual value of NDVI (NDVI) was obtained. Finally, the difference between the observed NDVI value (*NDVI_i_*) and *NDVI_i_* is calculated to obtain the NDVI residual value (ε_i_), and its trend rate (*Slope_εi_*) can be used to reflect the influence of non-climatic factors [42], such as human activities, on the trend of NDVI. The formulae for the multiple linear regression model and residual analysis are as follows:

(14) $$NDVI_{\mathrm{pre}}=\;a\times Tmp+b\times Pre+c+\varepsilon_{i}$$

(15) $$\varepsilon_{i}=\;NDVI_{i}-NDV{I^{\prime }}_{i}$$

where *a*, *b*, and *c* are the regression model coefficients; *Tmp* and *Pre* are the average temperature and cumulative precipitation, respectively; ε_i_ denotes the difference between the measured and predicted values of vegetation NDVI in year I; *NDVI_i_* denotes the measured NDVI value in year I; and *NDVI*′*_i_* denotes the predicted NDVI.

## 3. Results

### 3.1. Analysis of Spatial and Temporal Variability of Climate Factors

**Figure 2:**
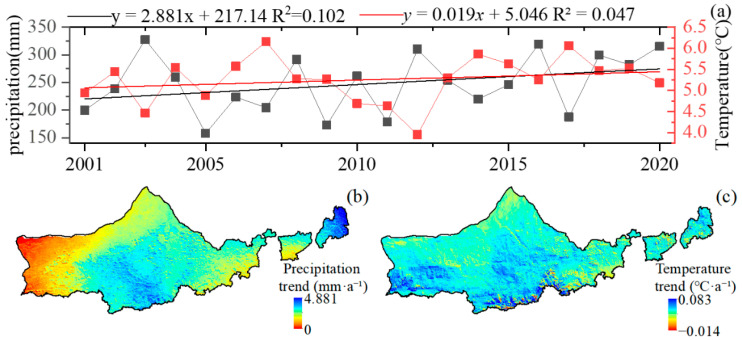
Temporal and spatial trends of Yinshanbeilu precipitation and temperature 2001–2020. (**a**) Interannual trends in atmospheric precipitation and temperature, (**b**) spatial trends in precipitation, and (**c**) spatial trends in temperature.

Water and heat are necessary conditions for the normal growth of vegetation, and climate change usually affects the growth of vegetation directly. In Figure 2, it can be seen that the climate of Yinshanbeilu from 2001 to 2020 shows a trend of “warming and humidification”. The annual precipitation generally shows an increasing trend, and the rate of change in annual precipitation is 2.881 mm/a, with a large overall change (Figure 2b. The places with a larger increasing trend are mainly located in Guyang County, Wuchuan County, and Duolun County (Figure 2a). The lowest annual precipitation occurred in 2005, with 166.68 mm, and the highest precipitation occurred in 2003, with 342.68 mm. The average annual temperature showed a fluctuating upward trend, with the highest average annual temperature occurring in 2007, at 5.98 °C, and the lowest average annual temperature occurring in 2003, at 4.3 °C, and the rate of change of the average annual temperature was 0.019 °C/a, showing a gradual increase from northeast to southwest (Figure 2b). This shows a gradual increasing trend from northeast to southwest (Figure 2c).

### 3.2. Analysis of Spatial and Temporal Variations in NDVI

**Figure 3:**
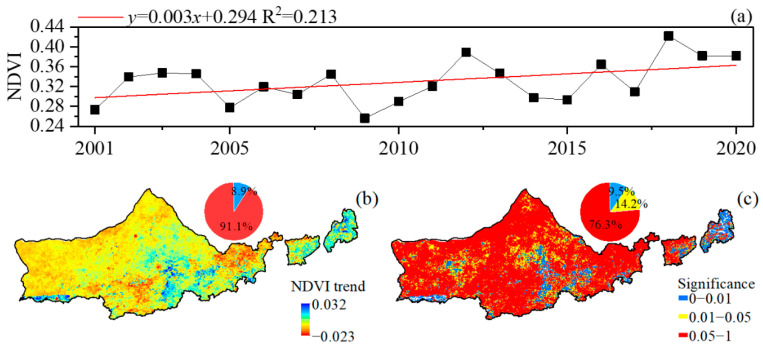
NDVI trend and significance. (**a**) Temporal trends in NDVI, (**b**) spatial trends in NDVI, and (**c**) significance distribution of NDVI spatial trends.

Over the past 20 years, the vegetation NDVI value has shown an overall upward fluctuating trend, from a 0.27 fluctuation in 2000 to 0.39 in 2020, with a growth rate of 44.45%, the average value over this period is 0.31, and the average growth rate of the rise is 0.003/a; the minimum and maximum NDVI values appeared in 2009 and 2018, respectively (Figure 3a), and the situation of the vegetation cover is improving.

The spatial trend of NDVI varied between −0.023 and 0.32/a (Figure 3b). Within this, 91.1% of the areas showed an increasing trend, and only 8.9% showed a decreasing trend (mainly in Shangdu and Huade Counties in the east). According to the significance test (Figure 3c), 76.3% of the regional NDVIs failed the significance test, only 23.7% of the regional NDVIs passed the significance test, of which only 8.6% showed a highly significant upward trend, and 0.9% of the regional NDVIs showed a highly significant downward trend.

**Figure 4:**
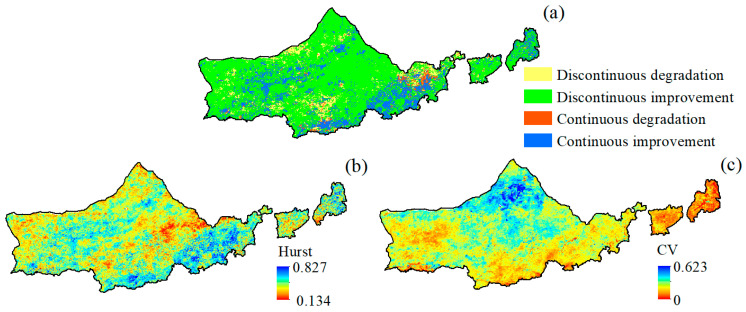
Characterization of spatial variation in NDVI: (**a**) future trend changes in NDVI, (**b**) Hurst index distribution, and (**c**) spatial variability.

The above analysis mainly reveals the spatial and temporal evolution characteristics of vegetation NDVI, while the future trend characteristics of vegetation NDVI are still unclear, so the image-by-image Hurst index of vegetation NDVI in Yinshanbeilu was calculated according to the R/S analysis method. The study showed that the Hurst index of vegetation NDVI ranged from 0.134 to 0.827, with a mean value of 0.593. The spatial difference of the Yinshanbeilu NDVI Hurst index was obvious, but most of the area had H > 0.50, with an area proportion as high as 87.36%, which was a strong positive persistence, indicating that the vegetation NDVI of most areas of Yinshanbeilu will still be in the future trend of vegetation NDVI to 2020 and afterward. This indicates that the NDVI of the vegetation in most areas will continue to rise after 2020. However, the proportion of the area with H < 0.50 was only 12.64%, which was spatially distributed mainly in the distribution area of elevated NDVI (Figure 4b), implying that the NDVI trend of the limited area of elevated NDVI in the study area would be changed from elevated to lowered sometime after 2020. In order to further analyze the future trend of NDVI, the Hurst index was superimposed over the NDVI trend to obtain the future trend of NDVI (Figure 4a). Combined with Figure 4a and Table 1, it can be seen that 18.08% of the areas with continuous improvement of future vegetation NDVI were mainly distributed in Wuchuan County, Chahar Right-Wing Middle Banner, and Chahar Right-Wing Back Banner in the south of Yinshanbeilu. Among them, the future vegetation NDVI sustained-improvement area in Shangdu is slightly larger than the anti-sustained-improvement (degradation) area, which is closely related to the ecological environment management project. The future vegetation NDVI anti-sustained-improvement (degradation) area accounted for 71.16%, which was mainly distributed in most of the northern part of Yinshanbeilu. Within this, the Damao Banner area has complex geomorphology, obvious soil sanding, and a more fragile ecological environment, and these areas may be degraded in the future.

Using the coefficient of variation to analyze the stability of vegetation NDVI in the study area, the NDVI coefficient of variation ranged from 0 to 0.623, and the spatial variability of NDVI was significant (Figure 4c), with the relatively stable area accounting for 78.45% of the whole area, and the unstable area accounting for 21.55% of the whole area. The high fluctuations were mainly distributed in the northern part of the study area including Siziwangqi and Damaoqi, where the vegetation was easily affected by external influences. The vegetation in these areas is susceptible to external influences.

### 3.3. Spatial Autocorrelation Distribution of NDVI Trends

The Moran’s I values (Table 2) of the NDVI trends of grassland, cropland, and meadow were all greater than 0 and passed the significance test, and the Z statistic values were 9.036–99.826, indicating that the spatial distribution of the NDVI trends of the different land-use types was not randomly distributed, and showed positive spatial autocorrelation. This demonstrated spatial agglomeration characteristics, among which the NDVI aggregation of the grassland was the most obvious, with a Moran’s I value for the grassland of 0.663, followed by the cultivated land and, finally, the forest land.

**Figure 5:**
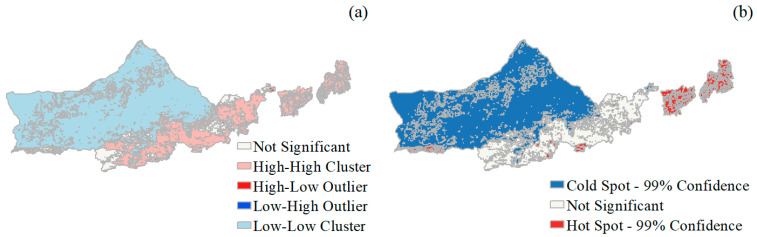
Spatially aggregated distribution of NDVI. (**a**) Localized Moran′s I value, and (**b**) Getis-Ord Gi* spatial distribution of NDVI trends.

As can be seen from Figure 5, the local spatial distribution of NDVI trends is characterized by high “band” and “block” clustering, low “block” clustering, and high and low “sporadic” clustering, accounting for 83.26%, 15.27%, and 1.47% of the total area, respectively. The proportions of the total area are 83.26%, 15.27%, and 1.47%, respectively. The high–high agglomerations are concentrated in the southern part of Yinshanbeilu, Wuchuan County, Chahar Right-Wing Middle Banner, Chahar Right-Wing Rear Banner, Shangdu County, Hade County, Taipusi Banner, and Duolun County, and the low–low agglomerations are mainly distributed in Yinshanbeilu, and the low–low agglomerations are mainly distributed in the southern part of Yinshanbeilu, and the low–low agglomerations are mainly distributed in the southern part of Yinshanbeilu. In the northern part of Yinshanbeilu, the low–low agglomeration area is mainly distributed in Ulatzhongqi, Damaoqi, and Siziwangqi. The high and low agglomerations are dispersed in localized areas around the low–low agglomerations, with the cold spot located in most of the northern part of Yinshanbeilu, and the hot spot in the southern part of Yinshanbeilu, in Taipusiqi and Duolun Counties.

### 3.4. Effects of Climatic Factors on NDVI

#### 3.4.1. NDVI Time Path Analysis

**Figure 6:**
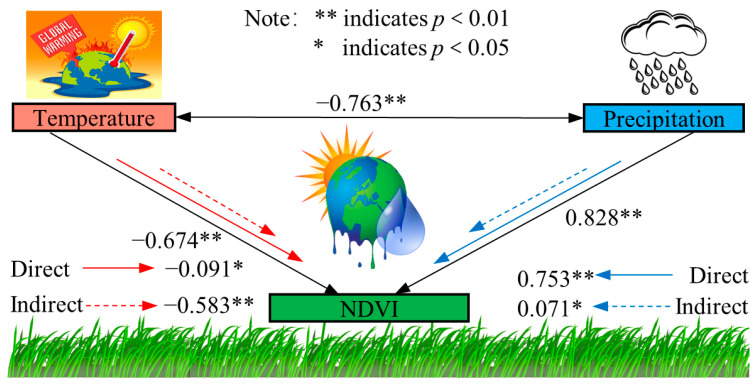
Schematic of NDVI pathway coefficients for precipitation/temperature.

Standardized multiple linear regression equations of temperature, precipitation, and NDVI had the statistic F = 27.359 (*p* < 0.01), and the process of pass-through analysis was valid. As can be seen in Figure 6, warming/cooling showed an inhibitory/promoting effect on vegetation growth (combined effect: −0.674, *p* < 0.01), and when the effect of precipitation changes was not considered, warming/cooling directly inhibited/promoted vegetation growth (direct effect: −0.091, *p* < 0.05), i.e., warming/cooling by decreasing/increasing precipitation had a greater effect on vegetation NDVI by reducing/increasing precipitation’s inhibition/promotion effect on vegetation NDVI by decreasing/increasing precipitation was greater than its direct promotion/inhibition effect on vegetation growth. Warming/cooling increases/decreases the atmospheric water demand, intensifies/weakens the water stress condition, inhibits/promotes photosynthesis, and is unfavorable/favorable to vegetation growth.

Without considering the effect of temperature change, increased precipitation can promote NDVI growth and significantly contribute to vegetation growth (direct effect: 0.753, *p* < 0.01). Also, increased precipitation would have a promoting effect on NDVI by decreasing air temperature (indirect effect: 0.071, *p* < 0.05). Overall, the correlation coefficient between precipitation and vegetation greenness was higher than that of air temperature, which had a significant effect on vegetation growth and was the main climatic factor affecting vegetation greenness.

#### 3.4.2. NDVI Spatial Path Analysis

**Figure 7:**
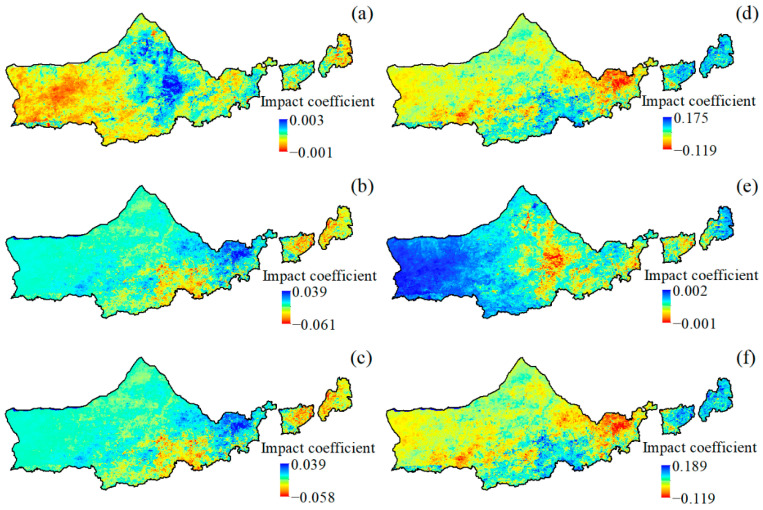
NDVI pathway analysis of precipitation/temperature. (**a**–**c**) represent the direct, indirect, and combined effects of precipitation on NDVI, and (**d**–**f**) represent the direct, indirect, and combined effects of temperature on NDVI, respectively.

The precipitation and temperature effects on NDVI of vegetation in Yinshanbeilu were spatially heterogeneous (Figure 7). The area is located in an arid and semi-arid region with high temperatures and low precipitation, and warming accelerates evapotranspiration, which dries out the soil and inhibits vegetation growth in most of the area. The majority of the northwestern part of the whole study is the concentration area of the positive influence, and the increase in precipitation will promote the growth of vegetation in most of the study area (Figure 7a–c). The vegetation in the southeastern part of the study area is relatively weakly affected by temperature and precipitation and includes areas of negative influence, especially the strongest negative influence of Duolun County and Taipushi Banner, which has a relatively good moisture condition, and the increased precipitation affects the light condition, weakens photosynthesis, and then inhibits the growth of vegetation. The increase in precipitation affects light conditions and weakens photosynthesis, thus inhibiting vegetation growth. The inter-annual variation of air temperature has a dominant inhibitory effect on vegetation NDVI, and the distribution range of the inhibition area is obviously wider than that of precipitation. However, the inter-annual increase in temperature can also lead to an increase in vegetation NDVI in local areas, mainly in the southern part of the study area, especially in the middle of the Right-Wing Banner of Chahar and the most concentrated in the back of the Right-Wing Banner of Chahar, which is at a higher altitude, and the heat condition is no longer the main factor limiting the growth of the vegetation. The warming will improve the photosynthesis of the vegetation and promote the decomposition of organic matter to improve the soil nutrients, which will promote the growth of the vegetation. The NDVI shows a promoting effect (Figure 7d–f).

### 3.5. Time Lag and Accumulation Effects of Climatic Indices on NDVI Change

**Figure 8:**
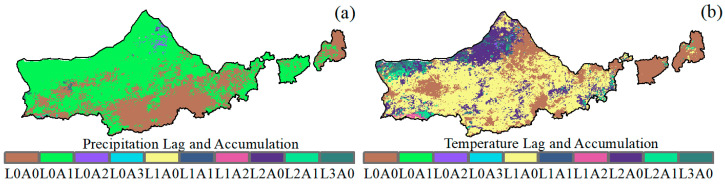
Temporal response of NDVI to climate factors. (**a**) Time lag and cumulative effects of precipitation on NDVI, (**b**) time lag and cumulative effects of temperature on NDVI. (LiAj means that the lag and accumulation months are *i* and *j* months, respectively).

Figure 8 shows the regional proportions for different combinations of lag and cumulative months. Under Lagacc, the mean lag and cumulative months of precipitation for vegetation growth were (0.94 ± 0.08) and (1.78 ± 0.13) months, respectively. The mean lag and cumulative months of temperature for vegetation growth were (1.63 ± 0.14) and (2.26 ± 0.18) months, respectively. The major combinations of rainfall lag and accumulation months were L0A0 and L0A1, which accounted for 18.45% and 65.39% of the total area, respectively. The main combinations of temperature lag and accumulation months were L0A0, L1A0, and L2A0, accounting for 12.31%, 53.25%, and 6.37% of the total area, respectively. Overall, the time lag effect of rainfall on NDVI was insignificant, but the time accumulation effect was more pronounced than temperature.

### 3.6. Impacts of Human Activities on NDVI

**Figure 9:**
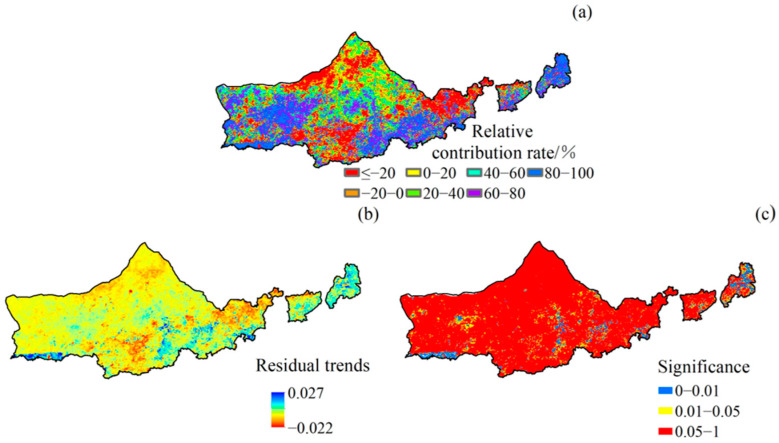
Impact of human activities on NDVI: relative contribution of human activities to NDVI (**a**), spatial distribution of NDVI residual trends (**b**), and significance tests (**c**).

The relative contribution of human activities to NDVI was characterized by significant spatial variability (Figure 9a). The area of the region with a positive contribution of human activities to vegetation NDVI change was about 77.04% (Figure 9b). Within this, the areas where the contribution of human activities was in the range of 40–60%, 60–80%, and 80–100% were larger, accounting for more than 20% of the area; the areas with a contribution of more than 80% were mainly concentrated in the eastern, southern, and part of the western part of Yinshanbeilu. The area with a negative contribution of human activities to the NDVI change of vegetation was about 22.96%, and its spatial distribution was similar to that of climate change. In most of the areas, the contribution of human activities to the increase in vegetation NDVI is generally smaller than that of climate change.

From 2001 to 2020, the trend of NDVI residuals of Yinshanbeilu vegetation showed obvious spatial variability, characterized by a high distribution in the south and a low distribution in the north (Figure 9b). Of these, the residuals of 72.25% of the area showed a positive trend, and 12.35% showed a significant increase; the residuals of 27.74% of the area showed a negative trend, of which only 2.68% showed a significant decrease, and the distribution was sporadic (Figure 9c).

## 4. Discussion

### 4.1. Characteristics of NDVI Spatial and Temporal Variations

Influenced by the regional climate differences, the NDVI of vegetation in the Yinshanbeilu area maintains the same distribution characteristics of high in the north and low in the south, and high in the west and low in the east, from 2001 to 2020. Studies have shown that moisture is the dominant factor limiting vegetation growth and productivity enhancement, and the warming and humidifying shift in the climate of Northwest China in recent decades has provided a more favorable growth environment for vegetation [43,44,45]. Temporally, vegetation NDVI showed an overall increasing trend from 2001 to 2020, with an increasing slope of 0.003 a^−1^, while the vegetation NDVI showed a significant decrease in 2009 and 2014, which was related to the major droughts that occurred in Inner Mongolia from 2009 to 2010 and in 2013 [46]. The drought caused a surplus and deficit of soil moisture on the one hand, leading to a decrease in the photosynthetic capacity of vegetation, and on the other hand, vegetation growth faced more severe water stress due to the acceleration of the evapotranspiration rate [47]. Spatially, 91.11% of the vegetation areas in the study area showed an increasing trend in NDVI, and there was a significant improvement in vegetation cover, among which Wuchuan County, Chahar Right-Wing Middle Banner, and Chahar Right-Wing Back Banner, which are mainly farmed, showed the most significant improvement, which is consistent with the existing studies [48], and further indicates that both natural and non-natural factors have an impact on the vegetation cover.

The NDVI response relationship with precipitation in the study area has obvious regional characteristics. Usually, in arid areas with low precipitation and high potential evapotranspiration, precipitation is one of the main limiting factors for vegetation growth; therefore, increased precipitation promotes vegetation growth. Whereas in Yinshanbeilu, where the vegetation type is mainly dominated by grassland, the fragility of its ecosystem leads to a weak saving capacity for rainfall, high surface evaporation, and the response of grassland to temperature is not as sensitive as to precipitation. Non-climatic factors such as human activities play a dominant role in the increase in the NDVI. On the one hand, the areas where human activities had a greater impact on the increase in the NDVI in this period were mainly located in the Middle and Back Banners of the Right-Wing of Chahar, where the land use type was dominated by cultivated land, and the progress of agricultural technology and the improvement of the management level (e.g., increase in the rate of irrigated agriculture, area of reclaimed land, pesticide use, and application of fertilizers) might be an important reason for the increase in the vegetative cover. On the other hand, the construction of the Three North Protective Forest System and the ecological project of returning farmland to forests and grasslands have been implemented in the study area since 2000, which has also had a positive effect on the increase in vegetation cover. Overall, the altitude of the study area is low; the highest altitude is about 1300 m, the temperature is less affected by the altitude change, and the overall temperature in the study area does not decrease with the increase in altitude, so when the altitude is high, the vegetation growth in the area is better, due to the factors of sufficient sunshine and abundant precipitation.

### 4.2. Effects of Climatic Factors on NDVI

Temperature and precipitation are the main climatic factors affecting vegetation growth, and the existence of interactions between the two makes their effects on vegetation growth more complex. In northeastern Yinshanbeilu, an increase in precipitation decreases air temperature to the detriment of vegetation growth [49], whereas in southwestern Yinshanbeilu, warming leads to a decrease in precipitation to the detriment of vegetation growth [50], indicating that air temperature and precipitation can have indirect effects on vegetation through interactions, which are rarely quantified, and that the through-traffic analysis method, which allows for the air temperature, the direct and indirect effects of precipitation on vegetation growth can be distinguished. In this paper, it was found that the warming of the study area is not conducive to vegetation growth, while the increase in precipitation promotes the growth of vegetation greenness, which is consistent with the results obtained by Liu et al. [51] using correlation analysis. Further research based on path analysis found that a Yinshanbeilu temperature increase would directly promote vegetation growth, but at the same time, it would change the precipitation, resulting in the closure of vegetation stomata and the evaporation of soil water and other phenomena, thus producing an inhibitory effect on vegetation growth, because the temperature increase’s inhibitory effect was greater than its direct promotion effect. The overall effect of warming on vegetation growth is inhibitory, which is consistent with the results of Fu and Sun [52], Li et al. [53], You et al. [54], and Lin et al. [55].

In this study, monthly NDVI was analyzed linearly with different combinations of time-lagged and cumulative months of climate variables. The time lag effect of rainfall on NDVI was found to be insignificant, but the time cumulative effect was more significant than that of temperature. Most of the major combinations of lag and cumulative months for rainfall and temperature were 0 months lag and 1 month cumulative and 0 months lag and 2 months cumulative. This result is consistent with the findings of Liu et al. [56] and Jin, et al. [57] on the temporal effects of average precipitation and temperature on vegetation change. Our study also found that the time accumulation effect contributed more to vegetation growth than the time lag effect. This may be due to the complex and nonlinear threshold of vegetation response. At the individual plant scale, an accumulation of rainfall and temperature is required to initiate the plant life cycle (i.e., seed germination, seedling growth, flowering). At the ecosystem scale, biogeochemical cycles that provide soil nutrients for plant uptake and growth also require cumulative temporal effects [58,59,60].

### 4.3. Human Impacts on NDVI

The spatial distribution details of the contribution of human activities to NDVI changes can be quantified by stripping out the impact of climate change on vegetation through the residual analysis method [61,62]. Yinshanbeilu has a complex topography and geomorphology, and there are obvious spatial differences in the natural environment, climatic conditions, socio-economic development, and the intensity and mode of implementation of ecological projects [63], which are reflected in the results of the residual analysis of the present study. This is reflected in the results of the residual analysis in this study, and the direction and intensity of the influence of human activities on NDVI changes showed significant spatial and temporal differences. The response of surface vegetation to ecological projects has a certain lag [64] and is also negatively affected by the development of urbanization. With the full implementation of the national ecological projects, large areas of sloping cultivated land and barren hills and wasteland were converted to forests and grasslands, and the effect of vegetation restoration gradually became obvious [65,66]; therefore, the human activities in the later part of the study period had a predominantly positive effect on the changes in NDVI, and the degree of the effect showed a significantly higher trend, which is basically in line with the results of Zhou et al. [67] and Zhao et al. [68].

### 4.4. Uncertainties and Limitations

At present, the spatial resolution of the large-scale study based on remote sensing images is low, and for the actual situation of localized areas, high-precision remote sensing images are still needed for deciphering. Also, for the many influencing factors within the image elements, there is no unified standard evaluation, and the identification of areas saturated with vegetation cover is still somewhat limited at present. In addition, multiple regression residual analysis has been widely used in the study of stripping out the contribution of climate change and human activities to vegetation. However, in the establishment of multiple regression equations, the meteorological elements selected by different studies are different, and in the attribution of human activities, it is not yet possible to explicitly quantify the impact of specific human activities, such as urban expansion, returning farmland to forests, etc., on changes in vegetation, which gives the results of the study a degree of uncertainty. Therefore, in the future, it will still be necessary to further refine the factors of vegetation change and the impacts of human activities. Combining technical means such as field surveys to improve the precision of the evaluation results will help to understand the internal causes of vegetation change.

## 5. Conclusions

(1) From 2001 to 2020, NDVI in the study area presented a significant upward trend, with an increase rate of 0.003/a. The vegetation coverage is generally good, 87.36% of the area shows strong positive persistence, and the stable change area is much larger than the unstable change area.
(2) The correlation between NDVI and precipitation is greater than that of temperature, with precipitation playing a major role in promoting NDVI, and temperature playing a stronger role in suppressing NDVI than in promoting it. Among these factors, the time lag effect of rainfall on NDVI was not obvious, but the time cumulative effect was more obvious than that of temperature.
(3) Climate change and human activities had a double effect on vegetation change, showing obvious spatial heterogeneity, and the contribution of human activities to the increase in vegetation NDVI was generally smaller than that of climate change, with relative contribution rates of 22.96% and 77.04%, respectively.

## Figures and Tables

**Table 1:** NDVI trend persistence statistics.

Slope	Hurst	Type	Ratios/%
<0	0 ≤ Hurst < 0.5	Discontinuous degradation	8.41
>0	0 ≤ Hurst < 0.5	Discontinuous improvement	71.16
<0	0.5 ≤ Hurst < 1	Continuous degradation	2.34
>0	0.5 ≤ Hurst < 1	Continuous improvement	18.08

**Table 2:** Global Moran′s I value for NDVI trends.

Type	Moran′s I	Z	*p*-Value
Grassland	0.663	99.826	0.000 *
Farmland	0.384	40.667	0.000 *
Forest	0.195	9.036	0.000 *

Note: * indicates passing the significance test.

## Data Availability

Not applicable.
